# Modernizing diagnosis of Alzheimer's disease: A review of global trends and Asia‐specific perspectives

**DOI:** 10.1002/alz.70536

**Published:** 2025-08-04

**Authors:** Takeshi Iwatsubo, Reisa A. Sperling, Alicia Algeciras‐Schimnich, Hidenori Arai, Anna M. Barron, Tammie L. S. Benzinger, Maria C. Carrillo, Christopher Chen, Seong Hye Choi, Igor C. Fontana, Jonathan Graff‐Radford, Joshua D. Grill, Judith Heidebrink, Chaur‐Jong Hu, Ryoko Ihara, Takeshi Ikeuchi, Atsushi Iwata, Fanny C. F. Ip, Fasihah Irfani Fitri, Clifford R. Jack, Jee Hyang Jeong, Jianping Jia, Nagaendran Kandiah, SangYun Kim, Hisatomo Kowa, Renaud La Joie, Yoshiki Niimi, Ryoji Noritake, Ozioma C. Okonkwo, Sebastian Palmqvist, Michael S. Rafii, Rema Raman, Yong Shen, Tanya Simuni, Heather M. Snyder, Orapitchaya Sriwannopas, Luke E. Stoeckel, Wiesje M. van der Flier, Huali Wang, Donna M. Wilcock, Henrik Zetterberg, Jin Zhou, Simin Mahinrad, Claire E. Sexton

**Affiliations:** ^1^ Department of Neuropathology Graduate School of Medicine The University of Tokyo Tokyo Japan; ^2^ Center for Alzheimer Research and Treatment Brigham and Women's Hospital Massachusetts General Hospital Harvard Medical School Boston Massachusetts USA; ^3^ Department of Laboratory Medicine and Pathology Mayo Clinic Rochester Minnesota USA; ^4^ National Center for Geriatrics and Gerontology Obu Japan; ^5^ Lee Kong Chian School of Medicine Nanyang Technological University Singapore Singapore Singapore; ^6^ Mallinckrodt Institute of Radiology, Neuroradiology Section Washington University St. Louis Missouri USA; ^7^ Medical & Scientific Relations Alzheimer's Association Chicago Illinois USA; ^8^ Department of Pharmacology Memory Aging and Cognition Centre Yong Loo Lin School of Medicine National University of Singapore Singapore Singapore; ^9^ Department of Neurology Inha University College of Medicine Incheon Republic of Korea; ^10^ Department of Neurology Mayo Clinic College of Medicine Rochester Minnesota USA; ^11^ Departments of Psychiatry & Human Behavior and Neurobiology & Behavior Institute for Memory Impairments and Neurological Disorders University of California Irvine Irvine California USA; ^12^ Michigan Alzheimer's Disease Research Center and Department of Neurology University of Michigan Ann Arbor Michigan USA; ^13^ Department of Neurology School of Medicine College of Medicine Taipei Medical University Taipei Taiwan; ^14^ Department of Neurology Dementia Center Shuang Ho Hospital Taipei Medical University Taipei Taiwan; ^15^ Department of Neurology Tokyo Metropolitan Institute for Geriatrics and Gerontology Tokyo Japan; ^16^ Department of Molecular Genetics Brain Research Institute Niigata Japan; ^17^ State Key Laboratory of Molecular Neuroscience and Molecular Neuroscience Center The Hong Kong University of Science and Technology & Hong Kong Center For Neurodegenerative Diseases Hong Kong SAR China; ^18^ Department of Neurology Faculty of Medicine Universitas Sumatera Utara Medan Indonesia; ^19^ Department of Radiology Mayo Clinic Rochester Minnesota USA; ^20^ Department of Neurology Ewha Womans University College of Medicine Seoul Republic of Korea; ^21^ Innovation Center for Neurological Disorders and Department of Neurology Xuanwu Hospital Capital Medical University Beijing China; ^22^ Dementia Research Centre (Singapore) & Neuroscience and Mental Health Programme Lee Kong Chian School of Medicine Nanyang Technological University Singapore Singapore; ^23^ National Healthcare Group Singapore Singapore; ^24^ Department of Neurology Seoul National University College of Medicine & Clinical Neuroscience Center Seoul National University Bundang Hospital Seongnam Republic of Korea; ^25^ Department of Rehabilitation Science Kobe University Graduate School of Health Sciences Kobe Japan; ^26^ Department of Neurology Memory and Aging Center UCSF Weill Institute for Neurosciences University of California San Francisco (UCSF) San Francisco California USA; ^27^ Unit for Early and Exploratory Clinical Development University of Tokyo Hospital Tokyo Japan; ^28^ Health and Global Policy Institute Otemachi Tokyo Japan; ^29^ Okayama University Kita Ward Okayama Japan; ^30^ Department of Medicine and the Wisconsin Alzheimer's Disease Research Center University of Wisconsin School of Medicine and Public Health Madison Wisconsin USA; ^31^ Memory Clinic Skåne University Hospital Malmö Sweden; ^32^ Department of Clinical Sciences Clinical Memory Research Unit Malmö Lund University Lund Sweden; ^33^ Alzheimer's Therapeutic Research Institute Keck School of Medicine of University of Southern California San Diego California USA; ^34^ Neurodegenerative Disorder Research Center and Brain Bank University of Science and Technology of China Hefei China; ^35^ Department of Neurology and the Institute on Aging and Brain Disorders The First Affiliated Hospital University of Science and Technology of China Hefei China; ^36^ Department of Neurology Northwestern University Feinberg School of Medicine Chicago Illinois USA; ^37^ Department of Internal Medicine Division of Geriatric Medicine Faculty of Medicine Ramathibodi Hospital Mahidol University Nakhon Pathom Thailand; ^38^ Division of Behavioral and Social Research National Institute on Aging Bethesda Maryland USA; ^39^ Alzheimer Center Amsterdam Neurology Vrije Universiteit Amsterdam Amsterdam the Netherlands; ^40^ Amsterdam Neuroscience Neurodegeneration Amsterdam the Netherlands; ^41^ Epidemiology and Data Science Vrije Universiteit Amsterdam Amsterdam the Netherlands; ^42^ Dementia Care and Research Center Peking University Institute of Mental Health (Sixth Hospital) Beijing Municipal Key Lab for Translational Research on Diagnosis and Treatment of Dementia National Clinical Research Center for Mental Disorders NHC Key Laboratory for Mental Health Beijing China; ^43^ Department of Neurology Center for Neurodegenerative Disorders IUSM/IUH Neuroscience Institute and Stark Neuroscience Research Institute Indiana University School of Medicine Indianapolis Indiana USA; ^44^ Department of Psychiatry and Neurochemistry Institute of Neuroscience and Physiology Sahlgrenska Academy at the University of Gothenburg Mölndal Sweden; ^45^ Clinical Neurochemistry Laboratory Sahlgrenska University Hospital Mölndal Sweden; ^46^ Department of Neurodegenerative Disease UCL Institute of Neurology Queen Square London UK; ^47^ UK Dementia Research Institute at UCL London UK; ^48^ Hong Kong Center for Neurodegenerative Diseases InnoHK Hong Kong China; ^49^ Wisconsin Alzheimer's Disease Research Center University of Wisconsin School of Medicine and Public Health University of Wisconsin–Madison Madison Wisconsin USA; ^50^ Clinical Development Neurology Eisai Inc. Nutley New Jersey USA

**Keywords:** Alzheimer's disease, Asia, biomarker, dementia, diagnosis

## Abstract

The landscape of Alzheimer's disease (AD) and related dementias (ADRD) diagnosis is evolving rapidly, driven by advances in disease understanding, biomarker tools, and disease‐modifying therapies. Modern diagnostic approaches emphasize biological precision, early detection, and dynamic frameworks that adapt to treatment‐induced changes in disease biology. These frameworks enable opportunities for personalized interventions—encompassing pharmacological and non‐pharmacological strategies—and for enhanced clinical trial design. However, implementing these advancements globally is influenced by diverse cultural, infrastructural, and regulatory factors. The 2024 Alzheimer's Association International Conference Advancements: Modernizing Diagnosis, held in Japan, provided a unique platform to explore these global dynamics, particularly from an Asian perspective. This article highlights key discussions from the conference, exploring the role of biomarker‐based diagnostic frameworks in shaping the future of AD/ADRD research, diagnosis, and treatment. We highlight regional challenges and successes and emphasize ethical considerations and practical strategies needed to ensure equitable access to diagnostic and therapeutic innovations.

**Highlights:**

- Advances in biomarkers are reshaping Alzheimer's disease diagnosis and treatment.
- Modern diagnostic frameworks highlight biological precision, early detection, and dynamic frameworks.
- The 2024 Alzheimer's Association International Conference Advancements: Modernizing Diagnosis explored challenges and opportunities in global biomarker implementation.
- The conference explored geographic‐specific impacts, focusing on Asia.

## 1 INTRODUCTION

The landscape of Alzheimer's disease (AD) and AD‐related dementias (ADRD) diagnosis is undergoing a transformative shift, fueled by a greater understanding of the disease processes, advances in tools to measure these changes through biomarkers, and the introduction of disease‐modifying therapies. Early and accurate diagnosis has become increasingly important, not only for empowering individuals and families with knowledge and planning opportunities but also for enabling timely intervention. This is especially true as anti‐amyloid beta (Aβ) monoclonal antibodies for the treatment of AD are entering clinical practice, which has demonstrated the greatest efficacy when initiated during the early stages of the disease.^1^, ^2^ In parallel, the past 2 years have seen the introduction of new diagnostic frameworks for some neurodegenerative disorders like AD, Huntington's disease (HD), and Parkinson's disease (PD) and Lewy body dementia (LBD; recently termed neuronal synuclein disease [NSD]) that are rooted in biomarkers, providing objective criteria for defining and staging these diseases.^3^, ^4^, ^5^ These frameworks have not only transformed research but are also bridging the gap between research and clinical practice, offering more biologically grounded tools for early detection, patient stratification, therapeutic decision making, and recruitment in clinical trials.

However, access to modern diagnostic tools is uneven around the world, with significant disparities between and within countries. In Asia, where rapidly aging populations and varied health‐care systems create unique challenges, efforts to modernize AD/ADRD diagnosis innovation can be accompanied by inequity. For instance, while there have been strides in integrating biomarkers and securing regulatory approvals for anti‐Aβ therapies for AD within Japan, China, and South Korea, other regions face hurdles such as limited health‐care infrastructure, low public awareness, and resource constraints.^6^, ^7^ The recent Alzheimer's Association International Conference (AAIC) Advancements: Modernizing Diagnosis, held in Tokyo, Japan, in September 2024, showcased these regional dynamics and challenges. The conference highlighted key achievements, including the development of biomarker research programs, regulatory approvals for therapies, and initiatives to improve clinical infrastructures.

In this article, we explore the evolving landscape of AD/ADRD diagnosis as discussed at the 2024 AAIC Advancements: Modernizing Diagnosis conference. With a focus on regional efforts within Asia, we discuss the integration of biologically based tools into clinical trials and clinical practice, as well as strategies for prevention. We also address the ethical and practical challenges of implementing these advancements, offering insights into the future of AD/ADRD diagnosis and care.

## 2 BIOLOGICALLY BASED DIAGNOSIS OF NEURODEGENERATIVE DISORDERS

A biomarker is a characteristic that is objectively measured and evaluated as an indicator of normal biological processes, pathogenic processes, or pharmacologic responses to a therapeutic intervention.^8^ In ADRD, clinically relevant biomarkers are generally categorized into biofluid‐based and imaging‐based markers. Biofluid‐based biomarkers, such as those found in cerebrospinal fluid (CSF) and plasma, are dynamic and reflect ongoing changes in the brain, capturing processes in real time. Imaging biomarkers, on the other hand, often capture accumulated tissue changes, representing the brain's structural or functional status. Notably, some imaging techniques capture synaptic function and neuronal activity, providing a bridge between static and dynamic insights.

Biomarkers in ADRD can also be categorized into markers that reflect the underlying pathophysiology of either protein aggregates, such as Aβ and tau proteins, or markers that identify tissue reactions to the aggregates, such as inflammatory and synaptic dysfunction. This distinction is essential, as protein aggregation (proteinopathy) and tissue reactions (such as astrocytic and microglial responses) play unique roles in disease progression.

In recent years, there has been a transformative shift in diagnosing neurodegenerative diseases, moving from symptom‐based approaches to definitions grounded in underlying biology. This transition reflects advancements in biomarker development and the growing recognition that many neurodegenerative disorders share overlapping symptoms. Within the past 2 years, new biologically based diagnostic and staging criteria have been introduced for AD,^3^ HD,^5^ and PD, and LBD (newly termed NSD).^4^ Here, we highlight some of these biologically based diagnostic criteria, underscoring the convergence in modern diagnostic and staging frameworks across these neurodegenerative diseases.

### 2.1 Convergence in modern diagnostic and staging criteria across neurodegenerative diseases

For most of the twentieth century, AD could only be conclusively diagnosed *post mortem*. This changed in 1984 when the National Institute of Neurological and Communicative Disorders and Stroke–Alzheimer's Disease and Related Disorders Association (NINCDS‐ADRDA) introduced the first clinical criteria allowing for a clinical diagnosis of probable AD based solely on clinical symptoms.^9^ The 1984 criteria made the crucial distinction between probable AD, which could be diagnosed in life, while definite AD could only be diagnosed at autopsy. Unfortunately, this crucial distinction was often ignored, and patients were given a diagnosis of AD based solely on clinical presentation.

Since then, advancements in biomarkers have transformed the diagnostic landscape of AD. In 2007, the International Working Group (IWG) introduced AD as a clinical‐biomarker construct and suggested integrating biomarkers into AD diagnosis.^10^ In 2011, the National Institute on Aging–Alzheimer's Association (NIA‐AA) workgroup published three separate recommendations for diagnosis of AD across its continuum—preclinical, mild cognitive impairment (MCI), and dementia—by incorporating biomarkers for diagnosis of symptomatic individuals.^11^, ^12^, ^13^ By 2018, the diagnostic model advanced further with the introduction of the A/T/N (amyloid, tau, and neurodegeneration) research framework (initially proposed in 2016^14^), which recommended defining AD purely by biological markers.^15^

In 2024, another workgroup supported by the Alzheimer's Association published the Revised Criteria for Diagnosis and Staging of AD. The Revised Criteria built upon the 2018 NIA‐AA research framework and newly includes plasma markers along with CSF and imaging biomarkers. Furthermore, the workgroup expanded criteria to encompass vascular, inflammatory, and other co‐pathological markers.^3^ The Revised Criteria classified biomarkers into three main categories: core biomarkers, biomarkers of non‐specific processes involved in AD pathophysiology, and biomarkers of non‐AD co‐pathologies. Additionally, distinct biological and clinical staging systems were proposed to capture the disease's complexity. The biological staging is a 4 point alphabetical scale (A–D) based on the ordering of biomarker events in the natural history of the disease, while the clinical staging adopts a 7 point numeric scale spanning from stage 0 (asymptomatic, deterministic gene) to stage 6 (dementia with severe functional impairment). The Revised Criteria also presented an integrated biological and clinical staging scheme. This scheme depicts the observation that the underlying pathophysiology of AD and its clinical manifestations are closely connected but are often not in lockstep: there are persons who exhibit relatively milder (or more severe) clinical symptoms relative to their disease burden. Taken together, the Revised Criteria present objective criteria for diagnosing and staging biological AD, moving the field closer to parlaying research insights into clinical care.^3^

Shortly after the publication of the 2024 Revised Criteria, the IWG responded with a similar proposal while arguing for a clinical–biological definition of AD. They noted that AD should only be diagnosed in symptomatic individuals with MCI or dementia, while a diagnosis of pre‐symptomatic AD could apply to asymptomatic individuals who carry very uncommon but highly penetrant genetic mutations.^16^ It is important to note that the Revised Criteria also emphasize that in the absence of approved treatments for asymptomatic individuals, biomarker testing for AD is not recommended in this population outside the context of observational or therapeutic research studies.^3^

In the PD and LBD field, the neuronal α‐synuclein disease integrated staging system (NSD‐ISS) Working Group has recently developed a data‐driven approach to define disorders characterized by neuronal α‐synuclein pathology and establish a staging platform.^4^ Within the NSD‐ISS framework, NSD is defined by the presence of pathologic neuronal α‐synuclein (S) assessed by a validated in vivo biomarker. Dopaminergic neuronal dysfunction (D), assessed by a validated in vivo biomarker, is the second core biomarker of NSD but is not required for the diagnosis. This biologic definition is designed to be independent of the presence of clinical features, or if present, of the specific clinical syndrome.

The NSD‐ISS integrates these biological anchors (S and D) with varying degrees of functional impairment caused by motor, cognitive, or other non‐motor signs. Individuals in Stages 0 and 1 are free of clinical signs and can be diagnosed either by the presence of fully penetrant pathogenic variants (G) in the *SNCA* gene (Stage 0), neuronal α‐synuclein (Stage 1A), or in combination with dopaminergic neuronal dysfunction (Stage 1B). Stage 2 is characterized by subtle clinical signs but no functional impairment, with either neuronal α‐synuclein alone (Stage 2A) or in combination with dopaminergic neuronal dysfunction (Stage 2B). Stages 3 through 6 require the presence of both biomarkers and a stage‐specific increase in the severity of functional impairment. This framework, applied in studies like the Parkinson's Progression Markers Initiative (PPMI) and intervention studies,^17^ provides a comprehensive biologically based framework essential to advance biologically targeted therapeutic development and is expected to evolve with emerging biomarkers.

These newly proposed biologically based criteria for diagnosis of neurodegenerative disorders share a number of foundational similarities, including (1) diagnosis based on biomarkers or a genetic marker, (2) recognition that each disease process exists on a continuum that begins years before the onset of symptoms, and (3) disease staging that integrates clinical symptoms with biomarker abnormalities.

Convergence on a biological diagnosis across the criteria for these neurodegenerative disorders is driven by the recognition that symptoms typical of each are not specific to that disease process. For example, progressive cognitive decline can be caused by diseases other than AD. Treatments targeting disease‐specific pathobiology require documentation of that pathology in patients participating in clinical trials or who are undergoing treatment clinically.

A second area of convergence across these neurodegenerative diseases is the recognition that the disease process begins years before the onset of symptoms. The disease exists and can be detected/diagnosed by biomarkers/genetic tests while affected individuals are asymptomatic. Extensive, irreversible damage is already present by the time symptoms occur. Therefore, one future goal should be identifying treatments that prevent or slow the onset of irreversible damage while patients are asymptomatic.

A final area of convergence concerns the structure of the disease staging schemes. The staging schemes for HD, AD, and NSD all lie on a 0 to 6 numeric scale. Stage 0 denotes individuals with a deterministic mutation who are asymptomatic and biomarker negative. Stages 1 to 6 require the presence of an abnormal biomarker. Stage 1 denotes a currently asymptomatic individual. Stage 2 denotes mild symptoms with no functional impairment. Stage 3 denotes mild impairment but preserved activities of daily living (this corresponds roughly to MCI in AD). Stages 4 to 6 denote progressively more severe functional impairment.

Taken together, these staging schemes anticipate a future in which more targeted treatment options exist, and staging will be used to match the individual patient to the optimal treatment approach either in trials or for clinical care. While these frameworks share foundational similarities, it is worth noting that they differ based on disease‐specific pathophysiology. These distinctions reflect the evolving understanding of each neurodegenerative disorder and their unique diagnostic and staging considerations.

### 2.2 Blood‐based biomarkers and blood proteomics in AD diagnosis

#### 2.2.1 Blood‐based biomarkers

Blood‐based biomarkers (BBMs) represent a significant advancement in AD diagnostics, offering a less invasive, cost effective, and more scalable alternative to CSF and imaging‐based diagnostics. These biomarkers have demonstrated promising diagnostic performance, with some assays now integrated as Core 1 biomarkers in the 2024 Revised Criteria for Diagnosis and Staging of AD. BBMs for AD are increasingly offered in clinical laboratories in the United States, although only those with a diagnostic accuracy > 90% align with the Revised Criteria.^3^ Among these, plasma phosphorylated tau (p‐tau) has emerged as a particularly promising biomarker. In 2020, several large studies demonstrated that plasma p‐tau is an accurate biomarker for AD.^18^, ^19^, ^20^ Since then, various assays have been developed and compared.^21^, ^22^ These comparisons show that p‐tau217 outperforms Aβ42/40, p‐tau181, and p‐tau231 for diagnosing AD, largely due to the significant increase in p‐tau217 levels in AD patients (2–7 times, depending on disease stage).

Comparative studies between mass spectrometry–based plasma p‐tau217 and US Food and Drug Administration–approved CSF biomarkers, using amyloid positron emission tomography (PET) as an outcome, show that higher‐performing assays achieve similar accuracy to CSF (≈90%).^23^ However, their effectiveness in clinical practice is closely tied to the pretest probability of AD. For example, in patients with a high clinical suspicion of AD, such as amnestic syndrome at the dementia stage, the test has a high positive predictive value (PPV) but may not be suitable for ruling out AD (lower negative predictive value). The reverse is true for those with low probability, such as subjective cognitive decline with non‐memory symptoms.^24^, ^25^

Composite biomarkers such as PrecivityAD2, which combines plasma p‐tau217 and Aβ42/40 ratios, have been also developed. This assay achieved 90% accuracy in primary and secondary care settings, significantly improving the diagnostic accuracy of primary care physicians (61%) and dementia specialists (73%) alike for correctly identifying clinical, biomarker‐verified AD.^26^ However, it is important to note that in this study, the amyloid positivity rate in the primary care cohort was ≈ 50%, which is higher than expected in most primary care populations. Further validation in some representative cohorts, particularly in settings with lower amyloid prevalence, is essential.

Despite these advancements, BBMs are not yet suitable as standalone diagnostic tools in clinical settings and should only be used as part of a full investigation, including cognitive testing. For example, while plasma p‐tau217 offers strong predictive capabilities for future AD dementia,^27^ a positive p‐tau217 result does not definitively indicate that AD pathology is causing the observed symptoms. To address this, emerging biomarkers, such as plasma MTBR‐tau243, which correlates with tau tangles, are under investigation. Such biomarkers could help differentiate between AD‐specific symptoms and other potential causes of cognitive impairment.

Innovative approaches are being adopted to integrate BBMs into clinical workflows. For instance, at the Mayo Clinic in the United States, the implementation of a clinical assay involved a direct comparison of two leading plasma p‐tau217 assays in individuals with MCI or early dementia. Particularly, the Fujirebio Lumipulse plasma p‐tau217 assay uses a two‐cutpoint model to categorize results as negative, intermediate, or positive. Using this approach, the combination of 92% sensitivity and 96% specificity to define lower and upper cut‐points provided the optimal balance between the number of false positives and false negatives, with < 20% of results falling in the intermediate range.^28^ Results in the intermediate range are then expected to have a confirmatory test such as CSF biomarkers or amyloid PET. This approach reduces the need for invasive or costly confirmatory tests by 84%. The real‐world performance of this assay continues to be evaluated by monitoring the percentage of intermediate results, which averages 18%. Additionally, several research studies are ongoing to further assess the concordance of this plasma p‐tau217 assay with amyloid PET and CSF AD biomarkers.^29^

#### 2.2.2 Blood proteomics and population‐specific considerations

Blood proteomic studies are also expanding the scope of AD biomarkers. The first large‐scale comprehensive blood proteome study, conducted in Hong Kong, quantified > 1100 proteins in the plasma of AD patients and healthy controls using a proximity extension assay. The study identified 429 dysregulated plasma proteins in AD, of which 19 were selected to form an AD blood signature biomarker panel. Based on this panel, a scoring system was established to distinguish AD patients from healthy individuals with > 96% accuracy and classify disease stages as normal, mild, or severe.^30^ Building on this foundation, a blood test for early detection of AD and MCI was developed. This test simultaneously measures 21 proteins associated with various biological pathways, achieving accuracy rates of > 96% for AD and 87% for MCI.^31^ Importantly, this test has been validated in different cohorts of Hong Kong Chinese and one cohort from Spain, demonstrating its applicability across different ethnic populations.

Different ethnic backgrounds exhibit distinct dysregulations in biomarkers, underscoring the need for personalized approaches in disease management. The study on Chinese and Spanish AD patients revealed significant variations in blood biomarker dysregulations across ethnic populations. These differences highlight the importance of considering that average differences based on race or ethnicity exist between populations in factors that influence AD biomarkers—for example, the prevalence of the apolipoprotein E ε4 allele. The mix of aging‐related neuropathologies that may contribute to cognitive impairment may differ on average by race or ethnicity. However, there is no evidence to suggest that the relationship between AD biomarkers and AD neuropathology is different based on race or ethnicity. Recent genome‐wide association studies (GWAS) further emphasize the need for ethnic‐specific references in blood biomarker development.^32^

The use of multi‐protein biomarkers not only enables earlier and more accurate diagnosis but also allows for stratification of patients by disease stage, which is critical in clinical trials and for treatment targeting. Additionally, these markers may reveal potential AD subtypes, which have been suggested through differing transcriptomic profiles,^33^ brain atrophy patterns, and clinical phenotypes.^34^ Multi‐protein panels can also identify subtle differences among these subtypes that traditional methods may overlook. Recent research indicates that medical conditions such as hypertension and diabetes can influence plasma biomarkers, including p‐tau and Aβ levels.^35^ Thus, using a composite biomarker panel may facilitate a comprehensive assessment of disease status and mitigate the impact of comorbidities on testing outcomes.

#### 2.2.3 Future directions

Co‐pathologies and comorbid conditions in AD individuals complicate clinical interpretation, underscoring the importance of combining BBMs with comprehensive diagnostic assessments. While there is currently no clinically actionable reason to incorporate blood tests into routine check‐ups for asymptomatic individuals, advancing research is crucial. In the future, if medically actionable steps become available, blood tests could play a role in routine screening. At present, BBMs are a powerful supplement but not a replacement for the comprehensive diagnostic process in AD. Moving forward, these biomarkers could significantly contribute to preventive strategies by identifying asymptomatic high‐risk individuals who could benefit from early interventions, potentially delaying AD progression and reducing societal and health‐care burdens.

### 2.3 Role of co‐pathologies in AD biomarker development

Some of the best‐established core biomarkers for AD include amyloid markers (Aβ42/Aβ40 levels in CSF and plasma; and amyloid PET), tau markers (p‐tau concentrations in CSF and plasma; and tau PET), and neurodegeneration markers (magnetic resonance imaging [MRI], CSF and plasma concentrations of neurofilament light chain [NfL] and brain‐derived tau). Together, these biomarkers can be used to stage the disease.^36^ Beyond these biomarkers, a growing body of evidence also highlights the importance of co‐pathologies. Clinico‐pathological studies have shown that 80% of older adults with probable AD have mixed pathologies, often with a combination of AD, cerebrovascular disease, Lewy body pathology, and TAR DNA‐binding protein 43 (TDP‐43) proteinopathy. This combination of pathologies suggests that AD is often not present in isolation, but rather as part of a broader spectrum of neurodegenerative and vascular conditions that collectively contribute to cognitive decline. However, the absence of validated biomarkers for many of these co‐pathologies complicates detecting and characterizing additional pathologies in vivo.

Cerebral small vessel disease, in particular, has been shown to accelerate cognitive decline when co‐occurring with AD pathology. Findings from the Religious Orders Study (ROS)—a large population‐based study in the United States—have demonstrated that cerebral small vessel disease can lower the threshold of AD pathology required to observe significant cognitive decline.^37^ In Asians, findings from the Biomarker and Cognition Study, Singapore (BIOCIS), a longitudinal cohort study of Southeast Asians from Singapore, demonstrated that ≈ 45% of participants with MCI have moderate to severe burden of cerebral white matter hyperintensities (WMHs), a surrogate measure of cerebral small vessel disease.^38^ WMHs were also demonstrated to have a significant negative correlation to performance in episodic memory, executive function, processing speed, visuospatial function, and language performance.^39^

Additionally, BIOCIS findings demonstrate a significant association between WMH and cerebral perfusion (measured using MR arterial spin labeling) and elevated levels of glial fibrillary acidic protein (GFAP), a marker of neuroinflammation.^40^ Using a novel non‐invasive imaging technique (DEBBIE), researchers demonstrated that increased blood–brain barrier (BBB) permeability is associated with increased arterial transit time, reduced gray matter perfusion, and higher WMH load. These results suggest that increased BBB permeability may be an upstream process in the deranged hemodynamics that contribute to the pathogenesis of WMH and that understanding the pathomechanism of WMH could allow for the development of novel biomarkers and intervention strategies for vascular cognitive impairment.^41^

As researchers uncover the role of additional pathologies in AD, biomarkers for other neurodegenerative pathologies are also advancing. Misfolded α‐synuclein seeds, detectable in CSF using real‐time seed amplification assay (SAA), are a promising biomarker for α‐synucleinopathies.^42^ While TDP‐43 biomarker development is still in its early stages,^43^ promising approaches include TDP‐43 loss of function–induced cryptic exon‐encoded peptides^44^, ^45^ or TDP‐43 concentration in plasma‐derived extracellular vesicles.^46^ Moreover, tissue reactions to neurodegenerative disease‐related proteinopathy—reflected in synaptic,^47^ astrocytic,^48^ and microglial activity^49^ markers—are increasingly recognized as essential biomarkers both using imaging measures and biofluid‐based tests.

Increasing evidence also supports that inflammation plays a significant role in AD and other neurodegenerative diseases.^50^ Genetic risk factors from GWASs have identified key immune regulatory genes, suggesting inflammation as a causal factor, not just a response to pathology.^51^ However, inflammatory biomarkers are challenging to interpret due to the diversity of microglial activation states—some of which are beneficial while others may be detrimental, depending on the disease stage. Therefore, a single inflammatory marker may not tell us much about the specific state of microglia in the brain.

The ultimate aim of biomarker research is to facilitate personalized medicine approaches for neurodegenerative disorders, identifying dominant pathogenic process(es) to tailor and monitor treatments aimed at preventing neurodegeneration.^52^ By delineating shared mechanisms across diseases, biomarker research holds promise for developing broad‐based therapies, which could revolutionize treatment for neurodegenerative conditions. The field continues to advance with promising new tools, such as PET ligands for α‐synuclein and TDP‐43, which, alongside fluid biomarkers, may offer a more complete view of these complex diseases.

### 2.4 Real‐world applications of biomarkers in clinical settings—case studies

While theoretical frameworks and population studies provide valuable overarching insights, the implementation of these concepts in clinical settings is often more nuanced and complex. Real‐world applications frequently reveal challenges that go beyond the theoretical frameworks, requiring tailored approaches to address individual patient needs. This complexity can be illustrated through case studies, which highlight the intricacies encountered in clinical practice.

With the approval of disease‐modifying therapies for AD, clinical workflows are evolving to integrate biomarkers in diagnosing and determining eligibility for anti‐amyloid therapy. This adaptation is necessary as a substantial portion of individuals with MCI may not meet eligibility criteria due to alternative underlying etiologies.^53^ Early clinical experiences with these new workflows have highlighted recurring themes around eligibility assessments for anti‐amyloid therapy, often revealing discordances between clinical presentations and biomarker results.

One recurrent scenario involves people who present with amnestic MCI and who have negative amyloid biomarkers. Often, these individuals display characteristics consistent with limbic‐predominant age‐related TDP‐43 encephalopathy (LATE), including age > 75, marked hippocampal atrophy that is disproportionate to other cortical regions, and fluorodeoxyglucose PET patterns demonstrating relatively isolated medial temporal hypometabolism sparing lateral parietal and temporal cortices, a pattern that contrasts with typical AD pathology.

Another scenario that has been encountered is the evaluation of patients with atypical presentations of AD for eligibility for therapy. For example, patients with logopenic primary progressive aphasia who appear more impaired on global cognitive testing due to their aphasia often perform well in activities of daily living, yet their Mini‐Mental State Examination (MMSE) scores may fall below the eligibility thresholds of clinical trials, leading to exclusion from treatment. Evaluating these individuals with functional scales such as the Clinical Dementia Rating (CDR) scale could offer a more comprehensive understanding of their functional capabilities and eligibility.

A third challenge arises with patients presenting features of mixed pathology. Patients with features of dementia with Lewy bodies (hallucinations, parkinsonism, cognitive fluctuations, or rapid eye movement [REM] sleep behavior disorder) may be amyloid positive > 50% of the time,^54^ leading to challenging discussions around eligibility. Obtaining additional biomarker support for an underlying synucleinopathy, through skin biopsy for phosphorylated synuclein^55^ or alpha‐synuclein CSF SAA testing,^56^ can aid in informed decision making.

Taken together, these real‐world situations often prompt challenging discussions on whether anti‐amyloid therapy is appropriate. Personalized assessments and shared decision making are essential as clinics are expected to increasingly encounter patients with complex, mixed, or atypical presentations. Below, we provide a summary of real‐world case examples from Japan presented at the conference, highlighting unique diagnostic challenges and approaches.

#### 2.4.1 Case examples in Japan

In Japan, clinical experiences with lecanemab have highlighted unique diagnostic challenges. In one example, a 74‐year‐old woman diagnosed with MCI at age 70 had been maintaining a stable MMSE score of ≈ 28. Her family, however, noted increasing memory problems, and she was brought to the hospital to be evaluated for lecanemab treatment. By then, her MMSE score had dropped to 24, though she scored 2 points on the delayed recall task, and her sense of time was accurate, which was somewhat atypical for an AD of > 3 years of history. There was no REM sleep disorder or visual hallucinations. During a consultation with physicians, fluctuations in cognitive function prompted a dopamine transporter scan (DaT) test, revealing a pattern of asymmetrical tracer loss consistent with LBD diagnosis. This case example illustrates the importance of recognizing the clinical characteristics of LBD and the early use of imaging biomarkers like a DaT scan for testing suspected LBD. With a prompt and accurate diagnosis, cholinesterase inhibitors like donepezil can provide effective long‐term management for these individuals.

In another case in Japan, a 59‐year‐old man presented with gradually progressive cognitive impairment, particularly affecting visuospatial ability. Although CSF biomarkers were negative for both amyloid and tau, flutemetamol PET scan revealed increased uptake characteristic of typical AD. A third case involved a 50‐year‐old man who presented with depressive symptoms with relatively preserved memory function. He was suspected of having AD due to asymmetric atrophy in the hippocampus and frontotemporal lobe on MRI. CSF testing was positive for amyloid and tau markers, but his Pittsburgh compound B PET scan did not show any amyloid accumulation.

These cases highlight discordance between amyloid PET and CSF Aβ biomarkers, a phenomenon that occurs in 10% to 20% of cases.^57^, ^58^, ^59^, ^60^, ^61^ Many large‐scale studies indicate that CSF Aβ biomarkers are more often positive than amyloid PET, particularly in the early stages of the disease or with certain mutations associated with autosomal dominant AD.^58^, ^59^, ^62^, ^63^ CSF‐positive, PET‐negative cases may reflect early fibrillar Aβ accumulation.^64^ Conversely, PET‐positive and CSF‐negative cases remain less understood. Differences in Aβ structure, individual microenvironments, CSF dynamics, or even preanalytical artifacts may contribute to these discordances. Using biomarker ratios rather than single values, along with procedural consistency, can help mitigate these inconsistencies. Ultimately, if a strong suspicion of AD exists despite a negative result from one biomarker test, it may be worth considering the complementary test(s) to confirm diagnosis and assess treatment eligibility.

## 3 TOWARD EARLIER DIAGNOSES: ETHICS AND OPPORTUNITIES FOR SECONDARY PREVENTION

Research increasingly shows that the AD pathology begins to change years before cognitive symptoms, marking a preclinical stage of the disease when biomarkers could measure these changes.^65^, ^66^ Identifying these biomarker changes is critical for early intervention strategies but has proven challenging, especially for those without a familial predisposition to AD—that is, sporadic AD. Although previous studies on biomarker trajectories have focused on autosomal dominant AD, these cases account for only a small fraction of overall disease cases.^67^ Furthermore, few long‐term studies have tracked longitudinal changes in AD biomarkers in persons with sporadic AD in Asian populations. A recent study from China aimed to address this gap, focusing on a diverse group of cognitively normal Chinese adults over two decades.

More than 1000 adults with objective cognitive impairment aged 45 to 65 were followed in the China Cognition and Aging Study for ≈ 20 years to examine long‐term AD biomarker trajectories. The study revealed that AD biomarkers diverged in a sequential pattern years before diagnosis: CSF Aβ42 levels at 18 years, Aβ42/Aβ40 at 14 years, p‐tau at 11 years, total tau at 10 years, NfL at 9 years, and hippocampal volume loss at 8 years. Cognitive decline showed significant differences beginning ≈ 6 years before diagnosis, highlighting critical early changes in AD progression. These findings provide insights into how biomarker trajectories unfold in sporadic AD among Asian populations. Understanding the temporal sequence and rate of these changes could help refine the design of clinical trials focused on preclinical intervention, potentially allowing treatments to be targeted more precisely at the earliest signs of disease pathology.^68^

Ongoing clinical trials, such as the TRAILBLAZER‐ALZ 3 and AHEAD studies, aim to prevent or delay cognitive decline by focusing on preclinical AD. TRAILBLAZER‐ALZ 3, which includes populations from Japan, Puerto Rico, Europe, and the United States, evaluates donanemab using a decentralized approach to improve accessibility. The AHEAD 3‐45 study examines lecanemab's effectiveness in preventing the cognitive decline associated with AD during the preclinical stage with targeted dosing based on amyloid levels at baseline, and is being conducted in Japan, Singapore, Australia, Spain, the United Kingdom, Canada, and the United States. Both trials contribute valuable data on the relationship between biomarkers and cognitive decline, offering potential breakthroughs in delaying AD progression during preclinical AD.^69^

Beyond pharmacological approaches, a paradigm shift in AD management emphasizes non‐pharmacological prevention strategies, particularly lifestyle interventions. For instance, the Dutch ABOARD project in Europe, a public–private partnership, aims to prepare for a future with personalized AD medicine through innovations in diagnosis, prediction, communication, and prevention.^70^ The World‐Wide FINGERS initiative focuses on multimodal interventions across 68 countries,^71^ seeking to replicate the findings of the FINGER study in Finland.^72^ In the Netherlands, the FINGER‐NL study is conducting a 2 year multidomain lifestyle intervention trial,^73^ recruiting participants through an online registry (Dutch Brain Research Registry^74^, ^75^) and using interventions that include lifestyle changes and nutritional support.

In Japan, J‐MINT demonstrated that a comprehensive approach combining exercise, nutrition, cognitive training, and social interaction can effectively support the maintenance and improvement of cognitive functions in older adults.^76^ Furthermore, the Japan Dementia Early Phase Project (J‐DEPP Study) was launched in 2024 to create a model for early dementia detection and intervention. This large‐scale study engages ≈ 10,000 participants from 36 municipalities, aiming to develop community‐level screening and support systems. The study starts with online screening tools for dementia risk, followed by comprehensive support and intervention for those with cognitive decline. Additionally, blood biomarkers, including Aβ, p‐tau, NfL, and GFAP, are measured to assess their utility in predicting dementia risk, as well as the predictive performance of the combination of blood biomarkers and cognitive function for diagnosing dementia. Those flagged as high risk are referred to primary care or specialized dementia care facilities for further evaluation. The study's framework not only enhances early detection but also serves as a sustainable model for promoting healthy aging.

Altogether, a proactive approach to AD prevention underscores the potential of identifying and modifying risk factors before dementia onset. With 14 modifiable risk factors now accounting for 45% of dementia risk, the challenge lies in tailoring prevention strategies to each individual and implementing these strategies effectively in public health and clinical care.

### 3.1 The ethics of preclinical diagnosis

Preclinical AD—also referred to as prodromal or asymptomatic—is defined as the biological presence of AD in the absence of clinical impairment. Formally proposed in 2011, the construct enables critical trials testing early intervention therapeutic hypotheses.^77^ The current secondary prevention trials anticipate a future clinical practice in which older individuals are screened for evidence of AD with biomarkers and begun on disease‐delaying treatments if indicated prior to clinical symptoms.^78^ Despite the growing use of the construct in research, most recommendations are against clinical testing for AD biomarkers in asymptomatic individuals,^79^, ^80^ including recent expert guidelines.^3^ The concern about translating the preclinical AD construct to clinical practice is because, at present, there are no disease‐modifying therapies available for people who are not yet clinically impaired.

Initial concerns about the safety of disclosing a preclinical AD diagnosis have not been realized,^81^, ^82^, ^83^, ^84^ though to date, this experience is limited to protocolized situations with substantial education and counseling. Access to this information may also have value for patients and families, enabling optimal financial, legal, and health planning.^85^, ^86^, ^87^ However, the risks of the preclinical AD construct include stigma and discrimination,^88^, ^89^ and few protections are in place to guard against them.^88^, ^89^ Moreover, despite substantial increases in the specificity of the preclinical AD diagnosis,^90^, ^91^ there remains uncertainty about the relative risk and timing of cognitive impairment among those meeting preclinical AD criteria, particularly among older persons who differ from those best represented in research studies.

When will this balance between risk and benefit tip toward clinical use? Most likely not until a treatment has been demonstrated as efficacious for delaying cognitive impairment in preclinical AD. Short of that, the field must pursue parallel advances in research and in the societies in which we live. Reduced stigma and increased access to resources to support patients on what will become a decades‐long journey will be essential to optimizing outcomes for older people living with the disease at all stages.^92^

## 4 OPPORTUNITIES FOR INTERVENTION

The recent regulatory approval of anti‐Aβ monoclonal antibodies is a major breakthrough for the treatment of patients at the early symptomatic stages of AD. In the Clarity AD study, lecanemab demonstrated significant improvements in biomarkers, and showed sustained treatment effects on the CDR Sum of Boxes (CDR‐SB).^2^ Similarly, the TRAILBLAZER‐ALZ 2 study demonstrated that approximately two thirds of participants treated with donanemab achieved amyloid clearance within 52 weeks, accompanied by a significant slowing of AD clinical progression as measured by Integrated Alzheimer's Disease Rating Scale (iADRS) score.^1^ The consistency of the effects across these two antibodies with slowing of clinical progression by ≈ 30% in the overall trial populations, with greater clinical benefit observed in subpopulations with evidence of lower amyloid or tau pathology at baseline, is encouraging for earlier interventions.

Such efficacy means that there is a need to better characterize patients who have achieved amyloid plaque clearance to guide clinical management and future trial inclusion. Convened by the Alzheimer's Association, a workgroup proposes the biomarker classification terminology termed treatment‐related amyloid clearance (TRAC). TRAC designations reflect that natural disease mechanisms have been altered and that there is biomarker evidence for amyloid plaque reduction. This terminology would be used to describe individuals who have (1) biomarker‐confirmed AD diagnosis prior to treatment, (2) received treatment with a drug that targets Aβ neuropathology in the brain, and (3) a follow‐up biomarker test indicative of amyloid plaque clearance, currently limited to PET. It is important to note that TRAC is a biomarker‐based classification: it reflects disease‐associated biomarker pharmacodynamic changes rather than direct neuropathological evidence. Also, it is fully expected that this framework will be adapted over time in response to rapidly evolving biomarker and clinical advances and with the accumulation of real‐world data on patients receiving anti‐Aβ monoclonal antibodies.

### 4.1 IMPLICATIONS FOR CLINICAL TRIAL DESIGN AND COMBINATION THERAPIES

The integration of biomarkers into AD diagnostic criteria has profoundly impacted clinical trials by enhancing the alignment of symptoms with underlying pathology. As a result, clinical trials are now better equipped to predict treatment responses, as well as the safety and tolerability of therapeutic interventions by looking at a biologically homogeneous sample. Furthermore, the standardized framework that these criteria provide enables earlier intervention, which is crucial for slowing disease progression in affected individuals. By operationalizing these criteria into clinical trial designs, the research community can effectively implement this new approach in moving earlier with greater phenotypic accuracy.

Given the current limits of amyloid‐based therapies alone in symptomatic disease, exploring therapies not related to amyloid remains essential. In addition, the advent of amyloid‐lowering treatment paves the way for combination trials. Multiple combinations of pharmacologic and/or non‐pharmacologic interventions are possible. The best combination will likely depend on the biological disease stage being targeted to match the evolution of processes that drive each stage. Combining amyloid and tau‐based therapies is a promising strategy and is being explored in DIAN‐TU Tau NexGen (ClinicalTrials.gov Number: NCT05269394) and the Alzheimer's Clinical Trial Consortium (ACTC) Alzheimer's Tau Platform trials.

An important consideration in designing these trials is the use of placebo controls. While amyloid‐lowering treatment as active controls or background therapies could enhance enrollment, they require adjustments to sample size, analysis methods, and biomarker‐based outcome assessments. A 2 × 2 factorial design offers a rigorous evaluation of combination therapies but demands a substantial sample size and requires the feasibility of being randomized to each of the interventions. To address these challenges, trials like the Alzheimer's Tau Platform have adopted a partial factorial design without a placebo group.^93^, ^94^

Taken together, biomarker advancements have revolutionized AD clinical trials, improving the alignment of symptoms with pathology and enabling more targeted interventions. Anti‐Aβ monoclonal antibodies have the potential for slowing disease progression, while new frameworks such as TRAC guide more precise treatment evaluations. The global shift toward early interventions and combination therapies highlights a promising future for AD research and patient care, with evolving trial designs poised to advance treatment efficacy and disease management strategies.

## 5 LANDSCAPE OF DIAGNOSIS IN ASIA

As populations in Asia age rapidly, countries across this region are facing increasing challenges in diagnosing and managing AD/ADRD. The diverse landscapes in this region reflect unique demographic profiles and health‐care systems, along with varying degrees of infrastructure, awareness, and resources dedicated to dementia care. In countries and territories like Japan, China, Taiwan, Indonesia, Thailand, South Korea, and Singapore, initiatives are underway to modernize AD/ADRD diagnostic approaches. These efforts span advancements in biomarker research, digitalization of health‐care services, policy reforms, and community‐based support initiatives. This section explores the current landscape of AD/ADRD diagnosis in these countries as discussed at the conference, highlighting the region‐specific challenges and developments that are shaping dementia care and early detection in the region.

### 5.1 Japan

Japan is the world's most aged society, with 29% of its population—≈ 36 million people—aged ≥ 65.^95^ This ‘“super‐aging” trend is projected to extend beyond Japan to other Asian nations such as China and Korea, which are also experiencing rapidly aging populations. In Japan, however, the older population has reached a demographic plateau, largely due to the aging of the baby boomer generation, which constitutes a substantial share of this age group. Over time, the number of older individuals in Japan is projected to gradually decline as this generation advances in age.

A critical issue arising from Japan's shifting demographics is the increasing proportion of older adults relative to a declining working‐age population. This trend presents a substantial challenge to sustaining economic productivity and supporting health‐care systems over the long term. To tackle this challenge, Japan will need to adapt its social and health‐care systems to operate effectively with a smaller labor force. Innovations such as task shifting, robotization, and digitalization of medical care delivery are key strategies to mitigate the strain on health‐care resources. Additionally, fostering enhanced collaboration between general practitioners and specialized care units is essential for optimizing care delivery.

A Japanese government report from 2014 projected that by 2025, 6.75 million people in Japan would have dementia. However, the latest 2024 estimates revised this number to ≈ 4.71 million, a reduction of nearly 2 million. This decline is likely attributed to healthier lifestyle choices, such as declining smoking rates. Despite this encouraging trend, government statistics from 2022 show that only 0.79 million AD patients were receiving consistent medical care, highlighting the need for improved health‐care availability and support structures in Japan. Addressing this care gap requires a multifaceted approach that builds a dementia‐friendly society. Key strategies include prioritizing early detection, providing community‐based care, and reducing the stigma associated with dementia. By fostering early detection and diagnosis and better integration of care at the community level, Japan can move toward improving the quality of life for individuals with dementia.

In Japan, advancements in AD treatment have accelerated with the recent approval of amyloid‐lowering therapies. Lecanemab, approved in 2023, and the pending approval of donanemab represent significant progress in targeting Aβ accumulation. Japan's latest dementia prevalence data projects a decrease in elderly dementia rates from 15% in 2012 to 12% by 2025^96^ attributed to lifestyle improvements, although rates of MCI are expected to rise. This suggests an emphasis on early‐stage AD interventions to prevent conversion from MCI to dementia.

The Basic Act on Dementia^97^ supports these trends, promoting an inclusive society in which individuals with dementia can express their abilities. The Act emphasizes public–private partnerships (PPPs) for fostering clinical trial environments, involving patients and families. Japan's trial‐ready cohort program, J‐TRC, aligns with this framework, providing web‐based and in‐person assessments to streamline recruitment for trials targeting early AD stages. Now in its second term, J‐TRC integrates PPP, public involvement, and biomarker‐based pre‐screening to support early and prodromal AD research.

### 5.2 China

Dementia affects ≈ 6% of the aged Chinese population, accounting for nearly one quarter of patients with dementia worldwide. However, systematic evaluation of AD using biomarkers, as outlined by the A/T/(N) framework,^15^ has been limited in China. Recent advancements in biomarker research, fueled by substantial funding from the Chinese government, have led to significant progress in understanding and identifying AD biomarkers specific to the Chinese population. Regional studies across China, including the China Cognition and Aging Study in the north of China (Beijing and Jinan), the Shanghai Aging Study in the east of China (Shanghai), the China Aging and Neurodegenerative Disorder Initiative (CANDI) in central China (Hefei), and research efforts in the central south China (Changsha), southwest of China (Chongqing), and south of China (Hong Kong), have used a variety of fluid and neuroimaging biomarkers. These studies highlight advancements in understanding preclinical AD stages, multimorbidity and cognitive decline, plasma biomarker applications, and the development of novel diagnostic tools across diverse populations in China.^68^ ^,^ 9^8^ ^–^ ^103^

China's biomarker research landscape is advancing rapidly, driven by robust government funding and collaborative efforts across diverse regions. The integration of biomarker technologies and longitudinal studies is enriching the understanding of AD in the Chinese population. These efforts help develop diagnostic and therapeutic approaches tailored to the unique genetic and environmental factors influencing AD in Asian populations.

### 5.3 South Korea

South Korea is experiencing rapid population aging, with > 20% of its population expected to be ≥ 65 by 2025. As of 2024, dementia prevalence is estimated at ≈ 10% among those aged ≥ 65 (≈ 1.2 million people), and this number is projected to increase significantly in the coming decades.^104^ In response, the government and medical institutions have actively implemented biomarker‐based diagnostics, research programs, and public health policies to modernize AD detection and care.

The National Dementia Screening Program (NDSP), launched in 2017, offers free cognitive assessments to individuals aged ≥ 60, promoting early detection of AD. Screening is conducted at public health centers and designated dementia centers across the country, with follow‐up evaluations available for those identified as high risk. South Korea has also made significant progress in integrating imaging modalities into clinical practice for AD diagnosis, adopting amyloid PET, tau PET, high‐resolution MRI, and artificial intelligence (AI)‐powered imaging analysis.

Among anti‐amyloid therapies for AD, lecanemab received regulatory approval from South Korea's Ministry of Food and Drug Safety (MFDS) in May 2024, while donanemab is under regulatory review. However, high costs and eligibility restrictions pose challenges to treatment accessibility. Efforts to improve access to disease‐modifying therapies include expanding South Korea's National Health Insurance Service (NHIS) coverage for anti‐amyloid therapies and reimbursement policies for diagnostic tests such as amyloid PET and CSF biomarker testing. These initiatives aim to facilitate broader access to innovative AD treatments while ensuring cost effectiveness and equitable distribution.

Additionally, a major national research study on dementia prevention is the SUPERBRAIN project, which integrates AI‐driven cognitive training, personalized exercise regimens, and dietary interventions to slow cognitive decline.^105^ SUPERBRAIN collaborates with leading academic and medical institutions to develop predictive models using multimodal biomarkers, aiming to identify individuals in the preclinical stage of dementia. This initiative aligns with South Korea's broader efforts to implement digital health strategies and precision medicine in dementia care.

Despite this progress, challenges remain. Plasma biomarkers need wider integration into routine clinical practice to reduce reliance on invasive CSF testing and costly imaging. Specialized dementia care is concentrated in urban centers, limiting access in rural areas. Furthermore, despite government‐led awareness campaigns, dementia stigma persists, often delaying diagnosis and care‐seeking behavior.

### 5.4 Indonesia

Indonesia, an archipelagic country with > 17,500 islands and a population of nearly 280 million, faces unique challenges in dementia care due to its demographic and geographic diversity. The country's aging population adds to the complexity of managing dementia. In the past 50 years, the proportion of older adults in Indonesia has increased from 4.5% to ≈ 10.7%, and it is expected to reach 20% by 2045.^106^ Estimates suggest that in 2019 there were 987,673 people with dementia in Indonesia,^107^ with local studies indicating prevalence rates between 20% and 29% among those aged ≥ 60 years, potentially exceeding global projections.^108^, ^109^ For example, a recent study reported a prevalence of 28% among 2110 people aged ≥ 65 years, suggesting that > 4.2 million people may be living with dementia in Indonesia, though formal diagnoses remain low.^110^ This high prevalence is partially attributed to increasing risk factors like hypertension, stroke, and diabetes.^111^ Consistent with this premise, a recent hospital‐based study in Indonesia indicated that vascular dementia was the most frequent type, likely due to the high burden of vascular risk factors.^112^

Despite the rising prevalence, dementia remains widely underdiagnosed partially due to stigma and low awareness. A study revealed that 86% of adults in Jakarta and North Sumatra were unfamiliar with dementia, often confusing it with normal aging.^113^ Other barriers to early diagnosis include time constraints in primary care and the lack of culturally appropriate cognitive assessment tools, in addition to different recommendations for cognitive tools.^114^ Recent efforts have focused on translating and adapting cognitive tests, like the Brief Cognitive Screening Battery, into Indonesian.^115^

In response to these challenges, Indonesia launched the Jaminan Kesehatan Nasional (JKN) program in 2014, aiming for universal health coverage.^116^ Within this scheme, provisions for dementia services are also present and governed, closely linked with other regulations and policies, including the National Dementia Action Plan. The dementia care pathway is structured across multiple levels, beginning with primary health centers that focus on screening and prevention, progressing to Type B hospitals for initial diagnosis and pharmacological management, and culminating in Type A hospitals equipped with advanced diagnostic capabilities, such as PET scans and CSF biomarker tests. However, rural areas face significant accessibility challenges due to limited resources and specialized facilities.

Addressing the dementia care gap in Indonesia will require a multifaceted strategy. This includes raising public awareness, improving access to culturally appropriate diagnostic tools, strengthening health‐care infrastructure, and supporting caregivers. Support systems, such as Alzheimer's Indonesia (https://alzi.or.id), play a crucial role in this ecosystem, offering education, promoting early detection, and providing essential support to patients and their families.

### 5.5 Thailand

As Thailand's population continues to age, the incidence of dementia is rising, underscoring the critical need for timely and accurate diagnoses to improve patient outcomes and quality of life. Addressing these challenges requires a comprehensive understanding of the dementia diagnostic landscape in Thailand, including health‐care policies, service accessibility, societal awareness, and the roles of various stakeholders. Currently, ≈ 680,000 adults over the age of 60 in Thailand are affected by dementia,^117^ and this number is expected to increase due to the aging demographic. AD and vascular dementia are the most prevalent forms, with younger‐onset dementia cases exceeding global averages.^118^ This trend highlights the necessity for effective diagnostic and intervention strategies.

Thailand uses various diagnostic tools for cognitive impairment assessment, such as the Thai Mental State Examination (TMSE) and the Montreal Cognitive Assessment (MoCA)‐Thai. These instruments have been culturally adapted to enhance clinical relevance. Diagnoses takes place in multiple settings including hospitals, which can provide diagnostic services in both public and private sectors; community health centers, which are critical for early identification and often serve as the first point of contact for those showing signs of cognitive decline; and specialized geriatric services, where focused teams deliver dedicated dementia care.

However, several barriers hinder effective diagnosis. A considerable lack of public and professional awareness about dementia leads many to overlook early cognitive decline signs, attributing them to aging. Limited access to trained health‐care professionals and diagnostic facilities, particularly in rural areas, results in misdiagnoses and delayed interventions. Additionally, traditional beliefs that equate cognitive decline with normal aging discourage families from seeking help and prioritizing familial care over professional assistance.

To tackle these challenges, Thailand has launched several initiatives. The Public Awareness Campaigns aims to reduce stigma and promote early diagnosis, supported by implementing a global dementia action plan using the Integrated Care for Older People (ICOPE) model. Technological innovations such as the AI‐based self‐screening tool “SAMONG” enhance accessibility to cognitive assessments. Training programs for health‐care providers are also being expanded, with collaboration among medical schools, the Alzheimer's Disease and Related Disorders Association‐Thailand (ARDA‐T), and the Thai Dementia Association to improve dementia detection in primary health‐care settings.^118^

Policy reforms have integrated dementia care into Thailand's national health‐care plan, with the Ministry of Public Health expanding services across 13 regions^119^ to ensure more comprehensive accessible care. In addition, Thailand's involvement in the WW‐FINGERS network^71^ promotes culturally relevant dementia research and interventions, further supporting the nation's efforts to enhance dementia care. By addressing these challenges through public awareness, health‐care training, policy reforms, and research, Thailand can substantially improve dementia diagnosis and patient outcomes.

### 5.6 Taiwan

In Taiwan, there are 0.35 million people living with dementia, and this number is projected to exceed 0.6 million by 2060. A 2023 report indicates that the prevalence of dementia among individuals aged ≥ 65 is ≈ 8%, consistent with data from a decade ago.^120^ Interestingly, there has been a slight decrease in prevalence among those aged ≥ 85, possibly due to the impact of the COVID‐19 pandemic, which disproportionately affected the elderly, especially those in institutional care.

In 2021, 296,000 dementia patients accessed outpatient services through Taiwan's National Health Insurance (NHI). Despite this, the number of new dementia diagnoses has not increased. This stagnation is likely attributed to a shortage of specialists and the long waiting times for annual neurocognitive testing, which are necessary for continued medication coverage. Medications such as donepezil, rivastigmine, galantamine, and memantine are covered by NHI, but the high demand for cognitive testing creates a bottleneck in the diagnostic process. To address these challenges, Taiwan has introduced computer‐assisted neuro‐cognitive testing to streamline the diagnosis process. This system prioritizes high‐risk patients for faster diagnosis and treatment initiation for those most in need.

In terms of diagnostic tools, MRI is covered by NHI making it accessible for many patients. Amyloid PET scans, however, remain self‐paid and costly, with only one tracer currently available in Taiwan. Efforts are underway to get NHI coverage of amyloid PET scans to reduce costs and increase accessibility. Additionally, Centiloid calculation software developed by Chang Gung University is available for amyloid PET scans, helping improve diagnostic precision. CSF biomarkers, using Roche Elecsys AD kit, are available but not widely used, and tau PET imaging is currently limited to research settings, though future commercialization is anticipated. Plasma biomarkers, using platforms like Simoa and immunomagnetic reduction (IMR), are also in research phases.

In summary, Taiwan continues to advance its diagnostic capabilities while addressing resource constraints to improve dementia care and outcomes. These advancements, alongside ongoing efforts to promote biomarker use and streamline diagnostic pathways, are crucial for managing the growing dementia burden in Taiwan.

### 5.7 Singapore

Singapore, a rapidly aging society, is experiencing a rising prevalence of dementia. Recognizing the importance of preventing age‐related diseases, the Ministry of Health has recently launched the Healthier SG program. This initiative aims to empower residents by providing an overview of key health parameters, setting personalized health goals, and creating actionable plans to achieve desired health outcomes and care preferences. Central to this program is focused on strengthening primary care and fostering partnership within the community care system.

The diagnosis process for early AD in Singapore typically involves cognitive testing, bloodwork, and imaging. However, testing for Aβ pathology is infrequently used due to the high cost of amyloid PET scans and the invasive nature of lumbar punctures. Consequently, biomarker confirmation of AD is often confined to research or clinical trial settings within academic institutions. Furthermore, those with early AD face challenges due to a limited number of dementia specialists and psychologists, particularly for the assessment of early dementia and MCI. The high cost of diagnostic biomarker tests as well as disease‐modifying treatments presents a significant barrier to widespread adaptation.^7^

Nevertheless, Singapore remains committed to becoming a “City of All Ages,” aiming to support seniors to live independently in their homes and communities. This commitment is reflected in the government's investment in senior‐friendly infrastructure, including housing, transportation, recreational facilities, and age‐friendly environments. Furthermore, significant funding has been allocated to address aging‐related issues, including dementia.

As part of this initiative, Phase 2 of the National Innovation Challenge on Active and Confident Aging prioritizes enhancing early detection and diagnosis of cognitive impairment and dementia. Ongoing and planned studies include the Digitised Cognitive Testing in Community Health Posts and Primary Care as well as the Blood biomarkers in Memory Clinics and Primary Care.

Singapore has also contributed to pivotal trials for disease‐modifying treatment in early AD and continues to participate in further trials targeting other causes of dementia. Additionally, the country is collaborating with other Asia‐Pacific nations to develop a robust framework for best practices and preparedness for advancements in the treatment of early AD.^6^, ^7^ These efforts are part of Singapore's broader strategy to improve dementia care and support systems, ensuring that its aging population can continue to thrive in a supportive and inclusive environment.

## 6 CROSS‐COUNTRY COMPARISONS TO MODERNIZE AD/ADRD DIAGNOSIS

Modernizing diagnosis in AD/ADRD requires implementing sensitive cognitive and clinical measures across diverse populations and incorporating biomarkers as intermediate outcomes within population‐representative samples. These measurements are essential to achieving an accurate, inclusive diagnostic framework that reflects the needs of diverse populations. The modernization of diagnosis therefore emphasizes not only revolutionary tools and methodologies but also their performance and applicability across varied demographic groups.

In this context, NIA has prioritized advancing AD/ADRD diagnostics by developing tools that are more inclusive, representative, and precise. This includes efforts to modernize diagnostic approaches through cross‐national comparisons to inform measurement development and a focus on diverse population samples, addressing disparities in dementia diagnosis and care.

An example presented at the conference was the Health and Retirement Study (HRS)—a population‐representative longitudinal study of > 20,000 older adults, starting in their early 50s, and followed every 2 years.^121^ HRS is internationally recognized for its multidisciplinary approach, combining cognitive, biomarker, economic, and social data. This framework has been adopted by numerous countries, including China and India, facilitating harmonized—not homogenized—data collection that enables robust cross‐national comparisons. To enhance cognitive assessment specifically, the Harmonized Cognitive Assessment Protocol (HCAP) was developed to provide national estimates of MCI and dementia in diverse, representative samples.^122^

Key insights from HRS studies include the importance of harmonized data collection to enable robust comparisons across populations and the potential to identify diverse AD/ADRD biomarker patterns. Notably, HRS studies have shown that only half of dementia and MCI cases exhibit the canonical AD biomarker pattern (lower Aβ42/Aβ40 and elevated p‐tau181), suggesting the need for more targeted diagnostic approaches.^123^ This includes data relevant to populations disproportionately impacted by AD/ADRD. For example, in Black American older adults, Aβ pathology might have a smaller role in determining cognitive impairment than other factors such as co‐occurring chronic medical conditions (hypertension, diabetes), cerebrovascular disease, and sociodemographic and systemic factors, each of which has been found to contribute to racial and ethnic disparities in dementia diagnoses.^124^

## 7 SUMMARY

The evolving landscape of AD/ADRD diagnosis reflects remarkable advancements in biomarker research coupled with the advent of disease‐modifying therapies. Modern diagnostic frameworks now emphasize biological precision, early detection, and dynamic adaptability to treatment‐induced changes. However, the implementation of these advancements globally remains challenging, and is influenced by factors such as cultural, infrastructural, and regulatory disparities. Insights from the 2024 AAIC Advancements: Modernizing Diagnosis highlighted the potential of biomarker‐based frameworks in shaping the future of AD/ADRD diagnosis and treatment. At the same time, the conference underscored the unique challenges faced by regions in Asia, where rapidly aging populations and diverse health‐care systems create both opportunities and challenges for modernizing AD/ADRD diagnosis. Initiatives such as Japan's trial‐ready cohort programs, China's advances in biomarker research, and Singapore's community‐based interventions demonstrate the importance of tailoring diagnostic frameworks to regional contexts. Addressing disparities in access to diagnostic tools and integrating innovative technologies will be critical for enhancing early detection and improving care delivery throughout the region. As the field progresses, a global commitment to equitable access, interdisciplinary collaboration, and the development of culturally relevant diagnostic tools will be critical in realizing the full potential of biomarker and treatment advancements. Together, these efforts pave the way toward a future in which individuals at risk for AD‐related cognitive impairment or living with AD/ADRD can benefit from timely, accurate diagnoses and effective, personalized interventions, ultimately transforming outcomes on a global scale.

## CONFLICTS OF INTERESTS STATEMENT

M.C. Carrillo is a full‐time employee of the Alzheimer's Association; received support from NIA, and grants or contracts from NIA and CDC. As a full‐time employee of the Alzheimer's Association, all travel expenses are covered by her employer. Participated on a data safety monitoring board or advisory board of NIA and NINDS funded initiatives including ADSP. Reports the following leadership or fiduciary roles: GHR Foundation—board, and American Heart Association—Research Committee (unpaid, no longer active). Her daughter is a neuroscience graduate student at USC. S. Mahinrad is a full‐time employee of the Alzheimer's Association. C. Sexton is a full‐time employee of the Alzheimer's Association. I.C. Fontana is a full‐time employee of the Alzheimer's Association. H.M. Snyder is a full‐time employee of the Alzheimer's Association. Received grants or contracts from NIA and CDC. As a full‐time employee of the Alzheimer's Association, all travel expenses are covered by her employer. Participated on the data safety monitoring board or advisory board (all external advisory board) of NIA and NINDS funded initiatives including DISCOVERY AD and Microbiome AD/ADRD studies, and NACC EAB. She reports the following leadership or fiduciary roles: Health Research Alliance—board (unpaid, past); American Heart Association—research committee (unpaid); liaison—Brain Health Council, American Heart Association (unpaid); Women's Brain Health Committee—AARP (unpaid); CDMRP, DoD Alzheimer's and Related Disorders Committee—Chair (unpaid); and XPrize Judge (unpaid). Her spouse works for Abbott in an unrelated area. T. Iwatsubo has received honoraria from Eisai and Eli Lilly. R.A. Sperling has served as a paid consultant for AbbVie, AC Immune, Acumen, Alector, Apellis, Biohaven, Bristol Myers Squibb, Genentech, Ionis, Janssen, Oligomerix, Prothena, Roche, and Vaxxinity. She has received research funding from Eisai and Eli Lilly for public–private partnership clinical trials and receives research grant funding from the National Institute on Aging/National Institutes of Health, GHR Foundation, and the Alzheimer's Association. Her spouse, K. Johnson, reports consulting fees from Novartis, Prothena, Merck, and Janssen. A. Algeciras‐Schimnich serves on advisory boards for Roche Diagnostics and Fujirebio Diagnostics and received speaker honoraria from Roche Diagnostics. T.L.S. Benzinger has received grants or contracts from Siemens paid to her institution; consulting fees from Biogen** ($5,000–10,000), Eli Lilly, Eisai** ($5,000–10,000), Bristol Myers Squibb, J&J, and Merck; payment for CME activity from Medscape, PeerView, and Neurology Today; and travel reimbursement from Cedars Sinai Medical Center, Hong Kong Neurological Association, and the Alzheimer's Association. T.L.S.B. reports the following patents planned, issued or pending: US patent 16/097, 457 (DIFFUSION BASIS SPECTRUM IMAGING (DBSI), A NOVEL DIFFUSION MRI METHOD USED TO QUANTIFY NEUROINFLAMMATION AND PREDICT ALZHEIMER'S DISEASE (AD) PROGRESSION), and US Patent 12,016,701 (Quantitative Differentiation of Tumor Heterogeneity Using Diffusion MR Imaging Data). T.L.S.B. has participated on a data safety monitoring board or advisory board of Siemens and served as an external advisor for NIH‐funded studies (no payments). T.L.S.B. has served as the co‐chair of ASNR Alzheimer's, ARIA and Dementia Study Group, and RSNA Quantitative Imaging Committee (QuIC) (all unpaid). T.L.S.B. has served as a committee member of the American College of Radiology/ALZ NET imaging, NIH CNN Study Section Chair, and had a leadership or fiduciary role in the ACR Commission on Neurology (all unpaid). T.L.S.B. has received technology transfer and precursors for radiopharmaceuticals from Avid Radiopharmaceuticals/Eli Lilly, LMI, and Lantheus, as well as a scanner loan from Hyperfine to her institution. For additional references regarding T.L.S.B. disclosures, see Sunshine ACT reporting here: https://openpaymentsdata.cms.gov/physician/850680. C. Chen has received grants or contracts from the National Medical Research Council of Singapore and has received support from the Alzheimer's Association for attending meetings and/or travel. J. Graff‐Radford has participated on DSMB for NINDS strokeNET; served as the site investigator for trials sponsored by Eisai and Cognition Therapeutics; has received NIH funding; and received payment or honoraria as a course director from the American Academy of Neurology and as a faculty from IMPACT AD, clinical trial course. J.D. Grill has received research support from NIA, the Alzheimer's Association, BrightFocus Foundation, Eli Lilly, Genentech, Biogen, and Eisai; has received travel support from the Alzheimer's Association and personal compensation for editorial service to *Alzheimer's & Dementia*. J. Heidebrink received grants from Biohaven, Cognition Therapeutics, Eli Lilly, Eisai, NIH/NIA; travel support from Alzheimer's Association to attend meetings; and honorarium as a faculty member for IMPACT‐AD clinical trials course (funded by grants from NIH and Alzheimer's Association). R. Ihara has received honoraria from Eisai, Eli Lilly, Fujirebio and Sysmex, PDR Pharma, Nihon Medi‐Physics, and IQVIA; participated on a data safety monitoring board or advisory board of Eisai, Eli Lilly, and MSD; and had leadership or fiduciary role in Japan Society for Dementia Research. T. Ikeuchi received grants or contracts from KAKENHI; consulting fees from FujiRebio, Eli Lilly, Sysmex, Eisai, and Novo Nordisk; and payment or honoraria from Eisai, PDR Pharma, FujiRebio, Kowa Pharmaceuticals, and Eli Lilly. A. Iwata has received advisory fees from Eisai, Eli Lilly, Biogen, Ohtsuka, Dream medical partners, Sound wave Innovation, and UCB; honoraria from Eisai, Eli Lilly, Biogen, Ohtsuka, Daiichi Sankyo, Sysmex, Fujirebio, Janssen pharmaceutical, MSD, and UCB; research grants from Eisai, Eli Lilly, Sysmex, Fujirebio, Fujifilm, SONY, MSD, Biogen, and Kobayashi Pharmaceuticals. F.C.F. Ip is a full‐time employee of Hong Kong Center For Neurodegenerative Diseases; received consulting fees from Cognitact Limited; has two patents licensed to Cognitact; and has stock or stock options in Cognitact Limited. C.R. Jack Jr. is employed by Mayo Clinic. He receives research grant funding from the National Institutes of Health, the Alexander family professorship, and the GHR Foundation. Within the past 36 months, he served on a DSMB for Roche pro bono; no payments to the individual or institution were involved. He has received funding from the Alzheimer's Association for travel to scientific meetings. H. Kowa received honoraria from Eisai and Eli Lilly. R.L. Joie consulted for GE Healthcare; received support for attending meetings and/or travel from the Alzheimer's Association; and grants or contracts from NIH, Alzheimer's Association, and US DoD. Y. Niimi belonged to the endowed chair; Department of Healthcare economics and Health policy supported by NN Life Insurance Company, Ltd.; and belongs to the endowed chair; Dementia Inclusion and Therapeutics supported by Effissimo Capital Management Pte Ltd. R. Noritake received consulting fees from Syneos Health Japan and AstraZeneca. O.C. Okonkwo reports grants from NIH; and leadership or fiduciary role in the International Neuropsychological Society. S. Palmqvist received grants or contracts from Avid Pharmaceuticals and Ki elements/ADDF (both paid to the institution); received payment or honoraria from Eli Lilly, Eisai, BioArctic, and Novo Nordisk for lectures, presentations, speakers bureaus, manuscript writing, or educational events; and participated on a data safety monitoring board or advisory board of Eli Lilly, Eisai, and BioArctic. M.S. Rafii received grants from Eisai and Lilly. He is a consultant for Ionis and AC Immune and serves on the DSMB for Biohaven and on the Scientific Advisory Board for Prescient Imaging, Positrigo, and Embic. R. Raman receives grant funding from the National Institutes of Health, Eisai, Alzheimer's Association, and the American Heart Association; travel support for participating in the Alzheimer's Association's managed conferences when a scientific committee member or invited speaker; is an emeritus Board member (unpaid) of Alzheimer's Association San Diego/Imperial Chapter. Y. Shen received grants or contracts and support for attending meetings/travel from the Chinese Academy of Sciences (XDB39000000) and Nature Science Foundation (31530089). T. Simuni has, in the last 12 months, served as a consultant for AskBio, Blue Rock Therapeutics, Critical Path for Parkinson's Consortium (CPP), Denali, Centessa, Genentech, MJFF, Prevail/Lilly, Roche/Genentech, Ventus, Sinopia, Ventyx, TrueBinding, and Vanqua Bio; has equity in Sinopia and has served on the ad board for Biohaven, GE, GAIN, Genentech, FDA, Neuron23, Parkinson Study Group, Prevail/Lilly and Roche/Genentech; has served as a member of the scientific advisory board of Koneksa and UCB; and has received research funding from MJFF, Neuroderm, NINDS, Parkinson's Foundation Prevail, Roche, and UCB. W.M. van der Flier has been an invited speaker at Biogen MA Inc, Danone, Eisai, WebMD Neurology (Medscape), NovoNordisk, Springer Healthcare, European Brain Council with all funding paid to her institution; is a consultant to Oxford Health Policy Forum CIC, Roche, Biogen MA Inc, and Eisai, all funding paid to her institution; participated in advisory boards of Biogen MA Inc, Roche, and Eli Lilly; is a member of the steering committee of phase 3 EVOKE/EVOKE+ studies (NovoNordisk), all funding paid to her institution; is a member of the steering committee of PAVE, and Think Brain Health; is a member of the Scientific Leadership Group of InRAD; was associate editor of *Alzheimer, Research & Therapy* in 2020/2021; is associate editor at *Brain*; is a member of supervisory board (Raad van Toezicht) Trimbos Instituut. H. Wang is a global advisory board member, Eisai Inc., and is served as president of the Chinese Society of Geriatric Psychiatry (unpaid). D.M. Wilcock received support for attending Alzheimer's Association International Conference (in 2022, 2023, and 2024) and AD/PD (in 2022, 2023, and 2024); participated on a data safety monitoring board or advisory board of BU‐ADRC EAB, Michigan ADRC EAB, UAB‐ADRC EAB, USC‐ADRC EAB, South Texas ADRC EAB, Phenotype Harmonization Consortium EAB, Fun‐Gen Consortium EAB, Synaps‐Dx SAB, Longeveron SAB, and Vigil Neuroscience SAB; serves as the editor‐in‐chief of *Alzheimer's & Dementia* journal. H. Zetterberg has served on scientific advisory boards and/or as a consultant for Abbvie, Acumen, Alector, Alzinova, ALZpath, Amylyx, Annexon, Apellis, Artery Therapeutics, AZTherapies, Cognito Therapeutics, CogRx, Denali, Eisai, Enigma, LabCorp, Merry Life, Nervgen, Novo Nordisk, Optoceutics, Passage Bio, Pinteon Therapeutics, Prothena, Quanterix, Red Abbey Labs, reMYND, Roche, Samumed, Siemens Healthineers, Triplet Therapeutics, and Wave; has given lectures sponsored by Alzecure, BioArctic, Biogen, Cellectricon, Fujirebio, Lilly, Novo Nordisk, Roche, and WebMD; is chair of the Alzheimer's Association Global Biomarker Standardization Consortium and chair of the IFCC WG‐BND; and is a co‐founder of Brain Biomarker Solutions in Gothenburg AB (BBS), which is a part of the GU Ventures Incubator Program (outside submitted work). J. Zhou is a full‐time employee of Eisai Inc. The rest of the authors have no disclosures. The content of this manuscript is solely the responsibility of the authors and does not represent the official views of the National Institutes of Health or the federal government. Author disclosures are available in the supporting information.

## Supporting information



Supporting Information
